# Kentucky State Dental Association

**Published:** 1861-06

**Authors:** 


### Proceedings of Societies.

### KENTUCKY STATE DENTAL ASSOCIATION.

The Kentucky State Dental Association held its second annual meeting at the Masonic Temple, in the city of Louisville, April 9th, 10th and 11th, 1861.

The first day was occupied in organizing, examining and admitting members, electing officers, and other preliminary business.

### WEDNESDAY MORNING, APRIL 10TH.

### *First Topic—Treatment of Temporary Teeth.*

Dr. J. W. GRANT, of Lancaster, by request of the President, opened the discussion.

The Doctor remarked that he had but little experience, except in the removal of the teeth at the proper time. In his own family had found his brothers' and sisters' temporary teeth failing quite early; he took the responsibility to treat and fill them, and thus kept them in the mouth and in a healthy condition until the permanent teeth were fully *developed*, thus preserving a full arch and a well developed and regular set of teeth.

Dr. PIERSON deems it of great importance to preserve the deciduous teeth until the proper time of shedding. Dentists should impress on parents the necessity of filling the temporary teeth as soon as decay commences. If requested to extract an aching tooth for a young child, invariably declines! Relieve the pain, destroy and remove the nerve, and fill the tooth. Has been very successful in the use of mechanical appliance, when the temporary teeth impede the development of the permanent, so as to produce irregularity. By means of gold spring and India rubber bands, sufficient expansion of the jaw may be obtained to permit the permanent teeth to be developed symmetrically, and thus avoid extraction.

His daughter, at the age of two years, complained of soreness in the left inferior molar. Examination and excavation showed a large crown cavity, with but a thin layer of dentine over the pulp—filled with amalgam, nicely prepared, and as free of mercury as possible. Five years have elapsed, and the filling remains apparently as good as the day it was put in. Have closely watched her teeth, and filled them as required. She now has nine teeth filled, but *has never known the toothache.*

The two inferior central incisors made their appearance at the lingual surface of the temporary teeth at five and a half years of age.

I let the temporary teeth remain until the fangs are absorbed, permanent laterals, lapped on lingual surface of temporary cuspidati, and stood at an angle of 35°. By removing the temporary cuspides out of the circle a little, the permanent lateral incisors became regular.

Dr. BALDWIN has had but little experience in that line; has occasionally treated and filled temporary teeth, with results similar to those of Dr. Pierson.

Dr. JONES treats such cases but seldom; in his portion of the country, but few cases of the kind fall under the notice of the dentist, but fully endorses what has been said.

Dr. LOCKERMAN has given much and careful attention to this subject, and attaches great importance to the preservation of the temporary teeth, let them be diseased as they may —treats locally and constitutionally, and retains them in the mouth as long as possible, even after abscess has formed; but regrets that his success in treating abscess has not been so great as could be desired—is preparing a lecture on this subject, to be delivered before the Kentucky School of Medicine.

Dr. SAUNDERS has nothing new to advance; but few cases come under his notice in this section of country; parents are not educated to the importance of the preservation of the temporary teeth—treats and relieves pain.

Dr. S. GRIFFITH—When deciduous teeth first commence to decay, before inflammation sets in, it is easily treated by removing decay, and filling. What is best to use is an important question. To use gold, the pressure must be considerable—more likely to induce inflammation of the periosteum, and ultimately abscess. Bone filling, when it can be used, is deemed best.

*Question*—Not in favor, neither will he fill a tooth where an alveolar abscess has been formed. Extracts in all cases where abscess has formed, but avoid extracting where it is possible, by the application of stimulants and sedatives. Lower molars the most important to be preserved as long as possible. Where a nerve has been exposed in such cases, has sometimes filled, but never satisfactorily.

Dr. Redman prefers saving the temporary teeth as long as possible, and keep the mouth healthy. Extracts only for irregularity. Little girl cutting permanent lower incisor, advanced half its length just back of the central and lateral. Extracted both the temporary teeth—removes only when they force the permanent out of the way—fills only with gold.

Dr. M'Clelland—Where a permanent lateral makes its appearance, should not be too hasty in desiring to see the teeth regular—they will generally regulate themselves.

Children are great sufferers from toothache, and the cases are difficult to diagnose; before probing decay, examine the stability of the tooth, so as to ascertain whether the pain is produced by concussion or occlusion of the teeth. Where a nerve is exposed, and aches only by contact of draughts of air, destroy nerve and fill. Where not exposed, cleanse cavity and fill with artificial dentine, as it requires but little pressure, and wears long enough. When the pulp dies without exposure, which is ascertained by percussion, cuts through without hesitation, generally finds nerve dead, or at least highly inflamed. Apply creosote and a little arsenic, and destroy the nerve, but fills only with cotton or something of that kind. Where the cavity is lateral, would fill.

Dr. Baxter—The development of permanent sets of teeth is vegetable crystalline—fills temporary teeth with lead in preference even to tin—they should be preserved from irritation or inflammation.

Dr. Griffith—After attentive listening, thinks we are all still very ignorant on the subject, and hopes it will have a position for next year, so it may be fully discussed.

Dr. Stone—After inflammation and suppuration have set in about the temporary teeth, there is no more absorption. Where the pulp is destroyed and the tooth dead, it becomes a foreign body—in such cases, always extracts. We may do injustice by extracting too early, and thus produce deformity—the object should be to protect patients from deformity, and at the same time save them from pain as much as possible. Where much irregularity presents itself, the first bicuspids on each side should be extracted.

Dr. LOCKERMAN—If a case applied of a child, tooth very sensitive, with cavity of decay, but nerve not exposed, would cleanse and fill.

Dr. STONE would not fill, but only cleanse with soap, removing all acid, and let it alone.

Dr. BAXTER wishes to know if the crown is filled, whether absorption ceases. Where does absorption commence? At the very apex of the fang, and as the fang absorbs, it gives crystalline form to the permanent tooth. Keep the crown healthy, and absorption will progress beautifully.

### *Causes of Caries, and Prophylactic Treatment.*

Dr. GRANT—This is a subject all dentists feel great interest in, still if we could discover a method of preventing decay, it would be a death blow to our profession. The causes of caries are found in chemical agents lodging between and around the teeth. Caries may be arrested by the judicious use of the file, judicious filling and treatment.

Dr. ROGERS felt some anxiety on this subject, and studied it some, and presented the subject to elicit information. The settled opinion is, that two causes produce decay—chemical and inflammatory. We often find on the buccal surface of the tooth abrasions of the enamel; the microscope shows this plainly, even when not visible to the naked eye. These crevices sometimes extend over the lingual surface, and upon the grinding surface; frequently the approaching edges of the enamel are so far apart as to fully expose the dentine; sometimes have cut this out and filled. In one instance found the tooth upon extraction split (both enamel and dentine) to the base. Cleanliness is the remedy.

Inflammatory causes must, for the most part, be presumed. Patient pointed out a spot on the tooth she suffered pain, but no decay visible. After frequent examinations, at the expiration of about three years decay appeared, thus showing that the chemical agents had not taken hold, but the decay had commenced inside. Another case, where tooth had been filled, decay appeared under the filling—can attribute it to no cause other than inflammation. Many cases show the pulp very far from the coronal surface, which shows the circulation to be feeble, and in such cases the dentine and the enamel are both frail.

Dr. Nourse has seen several cases similar to those described by Dr. Rogers, showing clearly to his mind that decay is frequently produced by inflammation, as well as by chemical influence. A patient, a short time ago, after frequent examination on repeated visits, finally discovered a dark spot under the enamel on the buccal surface, with no perceptible fracture in the enamel—broke the enamel, and found quite a large decay.

Dr. M'Clelland—Never have been a believer in internal decay; have broken a great many teeth, and never found a case of internal decay; inflammation will assist in the development of decay, in conjunction with chemical agents, both operating at the same time.

Dr. Griffith—One thing very certain, caries generally proceeds from the outside, he believes from chemical agents. Most frequently decay commences between the teeth, and is induced from the lodgment of food or other substances, which cause decomposition. Sometimes decay proceeds from exposure to extreme heat or cold. A lady had a tooth in which the nerve was destroyed by eating ice—drilled into the cavity, and pus discharged. These extremes produce inflammation, which induces internal decay. Stimulants and astringents to stimulate the gums, charcoal and Peruvian bark, are the usual and most desirable remedies, one as a disinfectant, the other a stimulant and tonic.

Dr. PECKOVER noticed twelve years ago all his children's teeth began to decay; took good care of them, and could not account for it; his wife has lost all her teeth, and he began to reflect whether the decay of the children's teeth was not inherited from the mother. Since then, he has almost in every case inquired of his patients as to the condition of the mother's teeth, and almost invariably received an answer that the mother's teeth were in a bad condition; therefore infers that the decay of teeth is generally constitutional. Children take their bony structure from the mother. Cold water and a brush for cleanliness, and corn bread for diet, are the best remedies.

Dr. DRIGGS—Can repeat only what is in the text books, and agrees with Dr. Peckover, with one exception, viz: that in those cases where teeth on one side were covered with tartar, found more cavities of decay on the opposite side.

Dr. GREEN—Decay proceeds from various causes—from the acids of the stomach and other chemical agencies, and from inflammation produced either by some concussion by blows, or from disease.

Dr. DWYER thinks the inflammation of decay is produced from chemical agencies, which reach the dentine through small crevices or abrasions in the enamel, which may not be visible to the naked eye, but still exist, and by breaking through with an instrument, large decay is apparent. These chemical agents are produced by acids from the stomach, eating candy, etc.

Dr. LOCKERMAN believes with Dr. Stockton—

> If when the teeth, designed for use,
> Decay, it is but from abuse.

In his observations, the result is different from Dr. Driggs—thinks he has never failed in finding less decay on the side of the mouth in constant use than on the opposite, which is covered with tartar and filled with decay, and from these appearances can without fail determine which side of the mouth is used; believes the dentist should instruct patients to chew all around; whether corn bread or corn beef, makes no difference; agrees with Dr. Peckover as to the constitutional or hereditary cause of decay.

Dr. TALBERT read somewhere that when a mother has very bad teeth during gestation, the child has bad teeth—wishes to know, if the teeth are extracted, whether the next child will have bad teeth.

Dr. REDMAN hoped to gain information. It is a daily question, why do our children's teeth decay so early? Causes of decay are very different, as is evident from the different character of the decay itself. Agrees with Dr. Driggs. Some patients can not keep the mouth clean; in some who are very cleanly, tartar will accumulate. Finds most decay on the side of the mouth most used.

Dr. LOCKERMAN—A gentleman applied yesterday; examined the mouth; uses the left side of the mouth, and no decay, while on the opposite side not a sound tooth exists, either above or below.

Dr. BAXTER can not attribute decay to any thing but chemical action or a congenital imperfection of the tooth, which exposes the dentine. Internal decay is a new thing—decomposition may take place, but supposes that to be chemical. The causes of decay are various, the most frequent are vitiated secretions, and this is made most apparent in women,—young mothers, the moment lactation commences. Decay also sets in, as nursing sore mouth. The best treatment is plain, unadulterated Bourbon whisky—wash the mouth with it, and drink it.

Dr. E. GRIFFITH has seen so many decayed teeth, under so many circumstances, and so many causes alleged, merely to show off a little extra knowledge; but as for any general cause, he is at fault, and it is equally difficult to name a specific cause. Some say mercury, some dyspepsia, and some give other causes—he himself has suffered for years with dyspepsia, and been frequently salivated, and yet his teeth are sound.

Dr. NOURSE, in explanation, remarked that from the appearance of the case before named, he was inclined to believe that internal decay might occur, and yet there may have been a slight fissure, but could not observe any—the tooth had been sensitive a year before any appearance of decay became visible.

Dr. GRANT spoke of chemical agents as the general causes of decay. Experience and rational deductions go to prove this to be the fact. Dr. Westcott, of Syracuse, has made a great many tests of food and fluids, that decomposition will take place in about eight hours, as suggested by Dr. Rogers, that enfeebled circulation would encourage decay—an entire suspension of circulation would destroy it. A lady, a patient of his father, lost several teeth by the use of ice. Hard food has a tendency to produce sound, strong teeth; animals that are fed on hard food have strong teeth, while those which are fed on soft food have inferior teeth.

Dr. M'CLELLAND—Cold will kill teeth; intense cold through metallic fillings will kill the nerve—the frequent application of vegetables, as pickles, lemons, etc., not only affect the stomach, but the teeth directly are injured.

Dr. W. D. STONE—You can not depend on hereditary causes alone for decay of teeth, neither can you calculate from the daughters or sons. His wife has bad teeth, and has had for many years, while all the children's teeth are good. Some of the children take after their father, and some after their mother; but if the mother's teeth are in a bad condition during gestation, there is no doubt but the child's teeth partake of the same character. Internal decay is impossible—any case presented to Dr. S. will receive $100 cash. Heat and cold can not produce decay directly—the jar or concussion of biting or chewing ice may produce inflammation, and thus the death; but suppuration will ensue, and will produce pain, and must have an outlet. Decay of teeth is mostly from external agents—either from food lying in contact long enough to produce softening, secretions or generation of acids or other chemical agents. Externel agents entering the fissures in the enamel, are the causes of disease. Cleanliness, being careful to keep away all foreign substances; if decay appears, apply at once to a good, honest dentist; have all the cavities or fissures filled; let the patient call on the dentist three or four times a year, and have the teeth examined. Cleanliness and plugging is the great secret of keeping teeth in good order.

### *Filling Teeth.*

Dr. GRIFFITH—The most important thing to make a filling valuable is to shape the cavity so as to retain a filling. Take an incisor tooth, central proximal decay; separate with a thin file, without fracture of the enamel, until space sufficient to work is obtained; use three or four excavators, straight stem or shaft, thin, hoe-shaped, cocked hat excavator. Remove all decay, cutting groove around the edge, and with a fine drill make pits or holes. If sponge gold is used, do so in small pellets. Gold foil in rolls, annealing in a spirit lamp after it is rolled; pack so as to let it project beyond the cavity; turn in the edges; pack with a fine-pointed instrument; dress with a fine file, and burnish.

Dr. TALBERT—We all prepare cavities alike. Almost five years ago commenced annealing in ropes, but found it difficult; since then anneal in the leaf, then make into ropes—use an instrument with a roughened point, consolidating each piece as you proceed—sometimes a small piece remains imperfect.

Take second molar lateral surface; with a hard drill made by cutting off an excavator and grinding up to an edge, excavate and drill holes into the corners—take Abbey's Foil, No. 6, one-third leaf; commencing in the lower edge, pack solid along from side to side, leaving the edge projecting; proceed in this way until the cavity is full; then with a small instrument pass around the edges carefelly, to find any points imperfect, if none such are found, considers the work complete. Dresses with a file, then with an Arkansas stone, pumice stone, etc., a stick and chalk, and then with a burnisher and soap, finish.

Dr. LOCKERMAN—Central incisor; first take separating file, cut on both sides, pass through—then a safe sided file, to get sufficient room—excavators need not be described. Shape cavity, cut retaining points; uses ropes annealed, and serrated instruments—commences at a single point on one side, and pack from side to side, condensing thoroughly as he goes, until the cavity is full, then with a sharp point, go all round the border, then condense the entire surface with a blunt point, and finish as usual.

Dr. PIERSON—Take a bicuspid; if teeth are close, and proximal surfaces decayed, take a chisel and separate; chisel, excavator and drill are the principal instruments. When the cavity is thimble-shaped, there is liability to slip; cut groove around the edge, and drill pits into the corners; use Watts', Abbey's or Leslie's gold foil; use serrated pluggers; anneal the foil. First fill the retaining points solid, then on this pack first; cut foil into strips of three or four to the leaf, according to size of cavity; roll into ropes, and cut the rope into squares; being sure the foundation is well laid, add piece upon piece. Commence at the center, and make it solid against the enamel, firm all the way, so close that no moisture can penetrate, dress with a file, then burnish with soap and a steel.

Dr. R. H. WILSON—Approximal surface of a central incisor above. First separate the teeth with either a file or chisel—prefer a sharp file—which he does freely; if with a chisel, follows with a smooth file, then with excavators—hoes, hatchets, etc. Removes decay from four retaining points, satisfied with two, if more can not be obtained; then introduce smal pellets of gold foil adhesive, prepared exactly as described by Dr. Pierson; fills the retaining points first; then with pellet after pellet builds up the filling to fill the cavity, condensing each pellet as he proceeds with sharply serrated plug gers. When the cavity is full, dresses with a serrated file, and burnishes with soap and burnisher.

Dr. BAXTER—Second molar, inferior posterior cavity. Seat patient; shield hand with a napkin, and pass into the mouth so the naked hand does not touch the mouth; then with curved files separates; press in a piece of pine close down to the gum, so as to prevent bleeding. With excavators remove the decay, making each cut count, but cutting as little above the pulp cavity as possible. With foil, cut into strips and rolled, take an instrument curved at angle of 75° or 80°, with a hook on the handle, so an assistant can use a mallet. Soft foil is used in bottom of cavity, followed with adhesive, using mallet all the time, until full; then file and burnish. The same process for an anterior cavity, using a straight instead of a curved instrument.

Dr. J. W. GRANT—First left inferior molar, decayed on the labial surface. If borders rough use chisel, to bring up the edges; then with right and left excavators removes decay, leaving the cavity largest inside or under cutting, particularly posteriorly—uses Abbey's gold foil, and with serrated instruments, with foil in ropes, pack towards the walls until the cavity is full and flush, then dresses and burnishes as usual.

Dr. M'CLELLAND makes some difference in the kind of foil used, whether above or below. Lower molar coronal cavity, embracing the approximal surface down to the gum. After cutting down the edge and removing decay, place a block in each side, extending out of the cavity, and fill between the block with adhesive foil, and so repeat until the cavity is more than full; then with burrs and a rolling motion, cut down to proper dimensions and polish.

Dr. REDMAN—Posterior approximal decay of second molar. Removes decay entirely with an excavator, cutting the cavity larger inside or under cutting. Then roll the strip of foil on an iron wire, so as to form a cylinder, using a mallet as large as can be without moving the gold; then condense; then place another but smaller pellet against one side of the wall; then another on the opposite, etc., using each pellet smaller than the former, until the last; then use as large a pellet and as solid as possible; then go all over it, with Baxter's plugging forceps, then fill and polish.

Dr. Saunders pursues a course similar to that of Dr. Pierson, but uses crystal gold foil with serrated pointed instruments; shape cavity, cut retaining points and pack with small pieces of foil, solid as you go.

Dr. Baldwin—Superior molar, grinding surface, large cavity. With chisel cut off thin edge; with hoe remove all decay, leaving the cavity a little larger at bottom than at opening; prepare gold (Abbey's); cut foil into three strips; wash out cavity and dry it; fill around cavity, and finish in the center; use instruments roughened on the sides for packing with serrated points. After all is full, double over the ends, and compress it; then with a sharp-pointed instrument feel all around, and if any imperfect points are found, force the instrument in and enlarge the cavity, which again are filled with small pellets—then with files dress to proper shape—with pine stick and pumice give surface, and finish with soap and brushes.

Dr. Nourse—Bicuspid approximal cavity, running into a coronal cavity. Remove all decay with excavators, and file the rough edges of the cavity; form the cavity, roll foil on a broach into block, the first as large as can be forced into the cavity; condense, and follow with strips, fold after fold, until the cavity is full, packing from wall to wall, finally finishing in the center. Fill up to proper shape, and burnish as usual.

Dr. Dwyer—Lateral incisor decayed up near the neck. Cut a groove on the inside to admit the instruments—drill small retaining points; wash and dry cavities; use foil No. 6, cut into small pieces (adhesive gold); with very delicate serrated pluggers pack first into those retaining points; fill up the cavity.

Dr. Driggs—Lateral incisor, lingual surface. Take a small drill, 1-16 in., using cutting motion to pass the drill into the cavity, cutting the cavity within a little larger than the outside; then with delicate drill make points towards the neck of the tooth; then a large burr drill is rotated in the cavity, to take off the sharp edges of the cavity; wash and dry the cavity, first with cotton, then with spunk; cut foil No. 4 into strips, anneal rolls into cylinders; then with pliers carry gently but firmly one into the cavity next the neck, the other towards the cutting edge; then follow with adhesive foil; fill the interstices, and pack full with serrated points; then with sharp cutting instruments cut the surface to the proper fullness; then with stick and pumice, and follow with Arkansas stone, and finish with soap and burnishers.

Dr. Greene—Crown cavity of molar; uses a drill to open cavity; uses a drill large enough to remove all decay, leaving a round, smooth cavity; after all decay is removed and cavity properly shaped, wash and dry; examine, and if any points of decay remain, remove it with a small drill; syringe and dry thoroughly; use French pluggers, with pellets, making a sure foundation; then build up pellet upon pellet until the cavity is over full; then file down, being sure that the filling is perfect around the margin, then polish with burnisher.

Dr. E. Griffith—Superior incisor, lateral approximal cavity. Separates, leaving a shoulder to prevent the teeth changing position; not make separation unnecessarily large in labial surface, filling from the lingual; remove decay with excavators and drills, using syringe from time to time throughout the operation, the better to be enabled to see what is doing. The rest of the operation is like others already described.

Dr. Stone—Left central incisor. Ten years ago filled approximal cavity between the centrals. Patient several times complained of uneasiness, but no discoloration appeared, but a sac was formed at the point of the root; removed filling, found nerve dead; fearing that could not pass through to the sac, took a fine nerve broach wrapped with a little cotton, and used it as a piston, until every particle of pus and dead nerve was removed; then again wrapped with fresh cotton dipped into creosote, so as to press in the creosote until it passed through and oozed out through the gum; dismissed patient, to return in three days. This was repeated every three days for two or three weeks, when all soreness and inflammation had disappeared. Then rolled gold on the point of a broach, and passed up as far into the fang as possible; then with another instrument followed, to force it to the apex —another pellet followed this, and so on until the cavity was full; then filled the crown, as described by Dr. Talbert. After so many years, saw the patient a few days ago, yet no recurrence of pain or inconvenience of any kind has appeared.

Dr. Rogers—Discoloration of teeth may be produced by chemical agents, absorbed by the gelatinous or cartilagenous portions of the tooth, also absorption of blood; disease of this portion of the tooth also produces discoloration. For this reason, insists on having complete control of the patients, in all cases where arsenious acid is to be used. Persevering use of chloride of soda has been successful in removing the discoloration.

Dr. E. Griffith thinks the absorption of blood into the dentine is the cause of discoloration—has seen cases where the color has been improved; but an entire restoration is out of the question.

Dr. Wilson—If arsenic is applied to a bleeding nerve, the tooth will in all such cases be discolored. To obviate this, the bleeding must be stopped before applying the arsenic.

Dr. Driggs has treated with chloride soda, but as he sees the patient only once a week, has met with but little success.

Dr. Jones cuts away until the discoloration is all removed.

Dr. Griffith—Discoloration is caused by being entirely cut off from all the vital forces, and this can not be restored, but if the external attachment of the periosteum continues, the discoloration is but partial, and a restoration may be effected.

Dr. Pierson treated many cases of discoloration, probably produced by absorption of blood and arsenic; uses 1 lb chloride of lime and 2 lbs. carbonate soda, and one gallon water. In other cases, uses chloride zinc confined by wax, and generally meets with success.

THURSDAY MORNING, APRIL 11TH.

*Extracting Teeth.*

Dr. SAM'L. GRIFFITH—Commenced at the dens sapientiæ; first cuts the gum freely on the posterior side with a right angle lancet, then on the sides—next take Griffith's elevator forceps, formed like a hawk's-bill splitting forceps; pass down to the process (the long beak always to the inside), and close gently, which will dislodge the tooth, and may be removed with a fang forceps.

For upper molars, use a forceps suited to fit the tooth, and with inward and outward motion break the attachments; but when he concludes to pull, does so outwardly, to avoid breaking the other teeth.

Bicuspids are extracted with the same parrot-bill forceps in the same manner as the dens sapientiæ.

Dr. LOCKERMAN—Not in condition to make remarks this morning. Speaking of the great Hayden, who asked him the question, "What is the ligamentum dentia?" in reply stated it was the periostic attachments.

In extracting any tooth, inserts lancet slowly to the process, passing it around slowly, so as to sever every attachment. Use for wisdom teeth a forceps similar to Dr. Griffith's, only bent differently. For molar teeth, uses Sherwood's forceps, which are better adapted to the different teeth than any other; must have a forceps to embrace the crown, as well as adapted to the neck; then with outward and inward motion, breaks the attachments, and lifts the tooth out. Bicuspids are treated in like manner. Cuspidata, in addition to the in and out motion, endeavor to give it a rotary. Incisors should always have the rotary motion.

Dr. NOURSE—Nothing new or peculiar; use the lancet thoroughly, and the proper instruments with the usual motion.

Dr. E. GRIFFITH discussed the modus operandi by which Caldwell extracted teeth. Lower molar, use elevator to dislodge, then pass forceps suited to the case; force up under the process until a hold is made upon the neck.

Dr. A. S. TALBERT spoke more particularly of molars when the crowns were broken off. Uses his ordinary forceps, but prefers a concave-pointed forceps, grasping at once the process with the tooth. If, however, it is broken so low as not to be within the reach of the forceps, use an elevator, bent at right angles upon itself, using the anterior tooth as a fulcrum.

Dr. GRANT uses Teft's local anæsthesia, to allay pain, and satisfy.

6 grs. morph. sol.
1 oz. chloride.
1 oz. aconite.

First separate gum with very narrow lancet, thinking it less painful than wider. Uses Sherwood's forceps; grasps with suitably shaped forceps (when the crown is frail) below the process, taking all out together.

Dr. JONES—Has nothing new; when he has difficult cases, "*gets them out*" in the best and easiest way possible.

Dr. WILSON—To extract the root of a central incisor which had been pivoted, introduces a screw to fill the cavity solid; then takes a forceps with straight, thin beaks, passes forceps as high up under the process as possible, and extracts with a rotary motion. When the root is so much inflamed and sensitive as not to admit the screw, uses a half conical pointed elevator, with sharp edges, passing it between the root and the adjoining tooth with a rotary motion; the oval back of the instrument serves as a fulcrum, while the sharp edge takes hold of the side of the tooth and rolls it out.

Dr. BAXTER—There is only one principle in extracting roots, that is, to get them out; if you can't one way, you must another.

The Doctor related an anecdote of a French dentist.

A vote of thanks was offered Dr. B. for the able and amusing manner in which he repeated the Frenchman's lecture.

Dr. Redman—Would like to have the sense of the meeting in regard to lancing the gums.

Dr. Pierson—In extracting roots of the superior central incisors and cuspidati, when decayed far below the process, I would recommend drilling with a small, sharp drill (suited in size to the point of the screw) nearly to the apex of the root; then follow with a burr drill of a taper width, and nearly as large as the screw. The carious part of the tooth is removed, and you have a cavity adapted to the screw.

Place the screw in the cavity, intervening it with care until it is firm, and you are confident the threads of the screw have cut into the bone of the root, giving it sufficient firmness, and the extraction will be performed with very little pain to the patient and much satisfaction to the operator.

Dr. Green uses Harris' improved forceps. Exhibited a molar tooth of rather peculiar form, taken out with one of those forceps.

Dr. M'Clelland—All are aware of the difficulty of extracting wisdom teeth. Even the shortest beaked forceps are generally too long.

In cases where the mouth can be opened but very little, say half an inch, the forceps is long in the joint and thin; in lower molars uses an instrument bent at right angles, placing the forceps on the tooth, pressing it firmly and slowly down.

Dr. Lockerman—Every thing has its objections. Unless a man is very filthy, looks upon tobacco as beneficial as a preservative. At times a soft calculi will collect between and around the teeth, which, if not removed, becomes hard. If a tobacco chewer is cleanly, the tobacco being frequently removed from between the teeth, the deposit will be removed with it. The constant friction of the teeth of tobacco chewers also prevents the accumulation of tartar.

Dr. M'Clelland—Tobacco, from its stimulating effect, causes redness of the membrane, also stimulates the glands to an excessive discharge of saliva. Nervous persons may be injured, corpulent ones benefited. This excessive secretion is of benefit to the teeth, and prevents the lodgment of particles of food or chemical deposits; we seldom find the ropy, viscid, acid saliva in the mouths of tobacco chewers. Where such saliva is present, much and rapid decay is apparent. Antiseptic properties are also possessed by tobacco, as will be found by old plates—the plate being of a more agreeable odor where tobacco has been used. It changes the character of decay from white to black.

Dr. NOURSE has nothing of much importance, but has observed the wearing away of the teeth by constant friction. Patients have stated that they are cured of toothache. On the whole, does not consider that tobacco has much effect on the teeth; it is very slight, but has no special effect on the health of the teeth.

Dr. E. GRIFFITH thinks tobacco has little effect on the teeth, not even in the wearing of the teeth, having seen many cases where the teeth are worn almost up close to the gum, but used no tobacco; and this is supposod to be no detriment, as such teeth seldom decay, much of the pulp cavity being filled with an osseous formation harder than the bone.

Dr. TALBERT thinks the use of tobacco is utterly incompatible with the character of our profession, as well as with the habits of a gentleman; its mechanical effect in the mouth certainly does, to some extent, prevent the deposit of tartar or any acids about the teeth, and thus to some extent prevents or arrests the progress of decay. Never recommend the use of tobacco under any circumstances as a preservative of the teeth; the injury to the system overbalances any supposed advantage.

Dr. GRANT has given the subject some thought, and is disposed to concur with Dr. M'Clelland. The effects are as various as the circumstances and constitutions of the persons using it. The mechanical effect is, doubtless, injurious, causing abrasion, wearing down the teeth, and causing the gums to recede.

Desires to hear from others on the use of snuff, or what is commonly called dipping. Thinks it very injurious in inflaming the gums and in denuding the necks of the teeth.

Dr. Baxter thinks tobacco has as many good as bad qualities—it is a good scavenger, fine shavings would do as well; directly, it does no harm, indirectly, its effects upon the secretions and general health are injurious.

Dr. Dwyer has experience—chewed many years, but abandoned it.

Dr. Redman—Chewed tobacco many years. Found it not injurious, but rather beneficial, lessening the sensitiveness of dentine. Thinks there is much difference in tobacco: has seen cases where it was evidently injurious, but believes it caused by something in the tobacco, not by the tobacco itself. The wearing of the teeth is caused by the peculiar way in which the teeth come together, as well as the density of the teeth and their peculiar antagonism—have seen many ladies with teeth worn down almost to the gum.

Dr. Lockerman suggested that the receding of the gums is produced by the use of snuff with a stick; the stick forces the gums away from the neck of the teeth, and the snuff forced under the free edge influences and causes a receding of the same.

Dr. Green—Used tobacco for a number of years; was induced to inaugurate the habit by having toothache, which was promptly relieved. Afterwards abandoned the habit, and resumed it again, but found the nervous system much affected; ceased and resumed several times, each time was the nervous system seriously affected. There are, no doubt, many who can chew with impunity, but others will seriously suffer in health.

### AFTERNOON SESSION.

### *Artificial Dentures.*

Dr. Sam'l. Griffith—Method simple; two modes to take impression—superior jaw full, takes impression in plaster, partial, in wax. Lower always in wax. Mix plaster with water, adding a little salt, place it in the mouth; can tell when it is set by the plaster remaining in cup; cut patterns in gold; take metal casts as accurately as possible, beat gold down into the female cast by pieces of wood, etc.; when well down, put on the male cast, and drive it home. This, however, does not fit, but with a duplicate cast swage again. Remove plate. Build up the wax and place it in the mouth; have the mouth closed; see that the contour is correct. Remove, and place in articulator. Grind in teeth, and solder. In partial sets makes suction plates wherever it is possible. Uses fusible metal, composed of bismuth, lead and block tin. Uses copper cup for investing and healing up for soldering.

Dr. DRIGGS—It is very important to begin right, to enable one to execute properly; therefore takes impressions in plaster for full dentures; for partial, first in wax, then in plaster. The plaster model is placed in a flask and heated; while hot, another ring is placed over the impression and filled with zinc. Separate and reverse, placing another ring, and pour lead on the zinc cast; thus you have the female cast. Plates being swaged, use air chambers, prefer to cut out plate and solder on another plate, thus giving a square edge, as well as being enabled to see if the plate fit there as well as elsewhere. Build up wax, place them in the mouth, mark the medium line, as well as several other marks, to enable placing together again precisely as before. Commence grinding with centrals, partially grind as far back as the bicuspids, which are generally found to stand perpendicularly; grind and arrange so that the molars and bicuspids will stand perpendicularly, even if the lower ones must incline very much inward to antagonize; finish grinding up close. The front ones must often be ground very thin. Arrange and sack the teeth, fit up and solder on the band, investing previously in sand and plaster, frequently remove the teeth from the investment, after soldering the rivets and dressing down nicely before soldering to the plate.

Dr. R. H. WILSON—Uses wax—the pine, plain country wax. Melts the wax into sheets about a quarter of an inch thick; then warm and soften before the fire before using,—careful to have sufficient to press with fingers closely over the arch, then around the edges. Remove from the mouth by pressure on the back muscles. Oil immediately, and pour plaster. As soon as the plaster is set, say six minutes, pour metal (8 parts bismuth, 5 parts tin, 4 parts lead), having first with a tack or pin attached to the plaster a piece of lead to form chamber, making a small hole entirely through the cast. Melt metal into an iron ring on a dry board, and dip the plaster cast while green; this promptly chills the metal around the cast, and makes it hug close. The metal is ready to pour just as it begins to granulate. Sift very fine whiting over the female cast, and form the male, of which take duplicates. Melt metal very slowly; if heated too hot, it becomes brittle. After cutting up plate, bends up plate over a series of iron dies, so that it nearly fits the cast. Before placing it on the cast, place it between the dies, and with a few heavy blows drives it home,—fits plates as high on the gums as possible, without interfering with the ligaments; forms a rim by rolling a strip of metal through the mill, one edge quite thin, leaving the other about as thick as a twenty-five cent piece. Builds up a wax form of teeth, tries in mouth, and changes until satisfactory. Grinds teeth, arranges with adhesive wax, replaces in the cast, cutting a groove around above the edge of the plate, so as to form a shoulder to fit band. Casts a metal cast on the plaster in two sections, divided in front, with duplicate metal; stick plate and solders in band, so as to let the end pass around the last molar, and soldered into the lining of the last molar, thus forming a continuous band all around, inside and outside. Uses an investment of sharp sand with plaster; heating up in a charcoal furnace—the cast fastened in a frame—with a bellows and gas blow-pipe, guiding the solder by a small pointed iron instrument, red hot, but not hot enough to burn; then places it in hot ashes to cool, and when nearly so, puts it in hot water, and thus cools off entirely ; then with lathe and burr cuts down the solder ; follows with scrapers ; then with pumice, rotten stone and oil wash, and finish it with rouge.

### *Miscellaneous Business.*

Dr. STONE—Destroys the nerve of teeth with a mixture of four grains of arsenic to one grain morphine, which must be thoroughly ground and triturated in a mortar, so it can never separate. Apply a very small quantity to the surface of the exposed pulp, which is left in the cavity from twelve to twenty-four hours, covered with a small roll of cotton. After with fine French nerve needles, with delicately barbed points, remove the entire nerve—the cavity is then injected with creosote until all bleeding and all soreness disappears. Then fill with gold to the apex of the fang, or as near to it as possible, the crown cavity being filled like any other tooth. If inflammation and neuralgic pain sets in, cut through the gum with a lancet to the process, and with a stronger sharp instrument cut through the process to the points of the fang ; then poultice with bread and milk poultice, so as to encourage suppuration.

Dr. PIERSON—Where the side of the face becomes so swollen that the petient could not see, nor could the dentist see the surface of the teeth, applied three leeches to reduce the swelling; treated with poultice and the wash of lime and soda.

Dr. REDMAN—In the case referred to, in inflammation at the point of the root, with much swelling, drill through the process into the cavity of the fang.

Dr. M'CLELLAND—In taking impressions under line, much difficulty occurs from the plaster coming against the cheeks and lips, also the dragging of plaster. To avoid this difficulty, a small instrument, called an impression fork, has been invented—it is made of silver, and introduced into the mouth while it is closed. Then spread out, so the patient has only to open the mouth, and the cup is passed in without touching the lips. After the plaster is set, the patient closes the lips tightly and fills the mouth with air. This loosens the plaster from the mouth; the fork is then passed astride the cup, and the cup is withdrawn without touching the lips.

6 Bismuth,
4 Lead,
4 Tin. In making dies, first dip in the ring as in the impression; use a compound metal, as per formula—proceed to make the male die first, direct from the plaster impression, and the female die is made from the male, of the same metal, which is melted slowly, and poured when nearly steady, to crystalize.

Dr. DRIGGS—Uses thick backing, and flows solder all over—when thick backing is used, splits rivets.

Dr. BALDWIN—Take impressions in plaster for whole sets, wax for partial; gets male cast of plaster; dips it into the metal while in the ladle; removes the female cast from ladle, lay in a ring, brushing all over with whiting and alcohol; then pour three or four male casts; drive up plate in cavities or holes, to get the shape of the cast; then hammer the plate up as near the shape as possible, after which strike up perfectly. Grind in teeth properly; then remove the teeth from the plate; lay them with rivets up in plaster and sand (white sand); use platina backs, and flow gold over the pins, of same quality as main plate. This enables to carry the backs up full to the point of the tooth, and drew up full and round, after which they are arranged again on the plate and soldered. Do not rim until all the teeth are soldered on, then oil teeth and plate; set into a ring and pour plaster around; remove the plaster without breaking. Place in position, and make a metal die, and with this swage the rim.

Dr. LOCKERMAN uses the metal described by Dr. Wilson.

